# A stacked machine learning-based classification model for endometriosis and adenomyosis: a retrospective cohort study utilizing peripheral blood and coagulation markers

**DOI:** 10.3389/fdgth.2024.1463419

**Published:** 2024-09-10

**Authors:** Weiying Wang, Weiwei Zeng, Sen Yang

**Affiliations:** ^1^ School of Pharmacy, Shanghai Jiao Tong University, Shanghai, China; ^2^ Shanghai Key Laboratory of Hydrogen Science and Center of Hydrogen Science, School of Materials Science and Engineering, Shanghai Jiao Tong University, Shanghai, China; ^3^ Department of Gynecology and Obstetrics, Shuguang Hospital Affiliated to Shanghai University of Traditional Chinese Medicine, Shanghai, China; ^4^ Department of Clinical Laboratory, Shuguang Hospital Affiliated to Shanghai University of Traditional Chinese Medicine, Shanghai, China

**Keywords:** endometriosis, adenomyosis, peripheral blood, coagulation markers, machine learning

## Abstract

**Introduction:**

Endometriosis (EMs) and adenomyosis (AD) are common gynecological diseases that impact women's health, and they share symptoms such as dysmenorrhea, chronic pain, and infertility, which adversely affect women's quality of life. Current diagnostic approaches for EMs and AD involve invasive surgical procedures, and thus, methods of noninvasive differentiation between EMs and AD are needed. This retrospective cohort study introduces a novel, noninvasive classification methodology employing a stacked ensemble machine learning (ML) model that utilizes peripheral blood and coagulation markers to distinguish between EMs and AD.

**Methods:**

The study included a total of 558 patients (329 with EMs and 229 with AD), in whom key hematological and coagulation markers were analyzed to identify distinctive profiles. Feature selection was conducted through ML (logistic regression, support vector machine, and K-nearest neighbors) to determine significant hematological markers.

**Results:**

Red cell distribution width, mean corpuscular hemoglobin concentration, activated partial thromboplastin time, international normalized ratio, and antithrombin III were proved to be the key distinguishing indexes for disease differentiation. Among all the ML classification models developed, the stacked ensemble model demonstrated superior performance (area under the curve = 0.803, 95% credibility interval = 0.701–0.904). Our findings demonstrate the effectiveness of the stacked ensemble ML model for classifying EMs and AD.

**Discussion:**

Integrating biomarkers into this multi-algorithm framework offers a novel approach to noninvasive diagnosis. These results advocate for the application of stacked ensemble ML utilizing cost-effective and readily available peripheral blood and coagulation indicators for the early, rapid, and noninvasive differential diagnosis of EMs and AD, offering a potentially transformative approach for clinical decision-making and personalized treatment strategies.

## Introduction

Endometriosis (EMs) and adenomyosis (AD) are both benign, estrogen-dependent chronic gynecological disorders (1, 2). EMs affects approximately 5%–10% of women of reproductive age (3), and the diagnosis rate is up to 50% among women seeking treatment for infertility (4). This condition is characterized by the presence of endometrial-like epithelium and/or stroma outside the uterine lining and muscular layer, often accompanied by associated inflammatory processes (5, 6). AD refers to a condition where the endometrial tissue infiltrates and grows within the uterine muscle layer, typically surrounded by hypertrophic smooth muscle cells and areas of fibrosis, forming diffuse or localized lesions on the anterior and/or posterior uterine wall (7). AD affects 19.5% of women of reproductive age (7). Both conditions can lead to dysmenorrhea, chronic pain, and infertility, severely impacting the quality of life of patients (6, 8). Although EMs and AD share similarities in their pathophysiology, their etiologies and clinical manifestations are significantly different (9). This implies that their treatment strategies and prognoses differ, necessitating that clinicians be able to accurately differentiate between the two for diagnosis. However, current diagnostic methods often rely on highly invasive surgical procedures and histopathological diagnosis (6, 10), which has led to a delay in the early differentiation of the two conditions.

In recent years, the application of machine learning (ML) technologies in the medical field has expanded significantly, particularly demonstrating tremendous potential in the areas of disease diagnosis and classification (11, 12). Stacked ensemble ML is a method that integrates multiple distinct models to enhance predictive performance and has been successfully applied in the classification and prediction of various diseases, offering new possibilities for noninvasive diagnostic approaches (13–16). With the increasingly important role of biomarkers in disease surveillance and diagnosis being recognized, the application of peripheral blood and coagulation markers in gynecological diseases has received extensive attention (17–25).

Using peripheral blood and coagulation parameters, this study developed a new classification method for EMs and AD using a stacked ensemble model. The highlights of this work are as follows:

(1) By utilizing a large retrospective cohort, we provided strong evidence for the effectiveness of the classification model presented in this paper, adding significant value to the existing methods for differentiating EMs and AD.
(2) This study was the first to apply specific biomarkers from peripheral blood and coagulation markers to the noninvasive diagnosis of EMs and AD, which could potentially reduce the need for invasive surgical procedures.
(3) A stacked ML model was applied to the differentiation of EMs and AD, integrating multiple distinct algorithms to enhance the accuracy of distinguishing between these conditions.
(4) The findings of this study may have paved the way for earlier and noninvasive diagnostic options for women suffering from gynecological conditions such as EMs or AD.

## Materials and methods

### Study design

This was a single-center, retrospective cohort study of consecutive women who presented with EMs and a comparative group with AD who attended Shuguang Hospital Affiliated with Shanghai University of Traditional Chinese Medicine (TCM). The diagnostic accuracy of the EMs and AD groups was evaluated based on a retrospective study design.

### Patient recruitment

Patients with EMs or AD were enrolled in the study through the Shuguang Hospital Affiliated with Shanghai University of TCM electronic clinical database. The recruitment process followed several steps to identify and select suitable candidates with complete and relevant data.

(1) Patient records: Potential participants with EMs or AD were identified by reviewing the medical records of patients who attended the obstetrics and gynecology inpatient departments at Shuguang Hospital affiliated with Shanghai University of TCM, between January 2016 and December 2023.
(2) Inclusion criteria: Patients with EMs or AD were identified through a retrospective review of medical records from the obstetrics and gynecology inpatient department at Shuguang Hospital Affiliated with Shanghai University of TCM, covering the period from January 2016 to December 2023. To ensure diagnostic accuracy, only those with a confirmed diagnosis of either EMs or AD, based on laparoscopic surgery and subsequent pathological examination, were included in the study. For each patient, only the laboratory and diagnostic test results from their first laparoscopic and/or pathological diagnosis were included in the analysis.
(3) Exclusion criteria: Women who had taken hormone medications within the three months prior to the study were excluded. Additionally, those with severe medical conditions, comorbidities, or acute inflammatory diseases that could confound the analysis were not included. We also excluded women with missing essential demographic details or incomplete routine blood test and coagulation function metrics.

### Independent variables

This study investigated the potential value of a comprehensive set of key hematological and biochemical parameters in the diagnosis and prediction of EMs and AD. Venous blood samples were collected from patients and analyzed using an automated hematology analyzer for complete blood cell counts, including red blood cell count, white blood cell count, and platelet count. Additionally, platelet indices and coagulation function markers were measured using chemiluminescence immunoassay techniques.

### Statistical analysis

In the statistical analysis that was conducted using SPSS version 26.0, the significance threshold was set at *α* = 0.05. Because the data in this study did not adhere to a normal distribution, the results are presented as medians with the 25th and 75th percentiles [M (Q25, Q75)], and the Mann‒Whitney U test was applied for intergroup comparisons. The Delong test was used to assess differences in the area under the curve (AUC) between the models. A *P*-value less than 0.05 (*P *< 0.05) was interpreted as indicating a significant difference between the groups under analysis.

### Feature selection

Feature selection helps remove irrelevant features to prevent overfitting. Feature selection was conducted before ML modeling to reduce data dimensionality, enhance model training efficiency and predictive performance, and improve the generalization ability of the model to new data (26). Within the EMs and AD groups, data demonstrating significant discrepancies underwent normalization via the *Z* score technique to mitigate variances across numerical scales. Three ML algorithms were used to screen for hematological feature factors: logistic regression (LR), support vector machine (SVM) classification, and K-nearest neighbors (KNN) classification. The specific parameters are detailed in Supplementary Table S1. LR classification measured the importance of feature variables through the coefficients obtained after L1 regularization. SVM classification and KNN classification both assessed the importance of features using the Recursive Feature Elimination (RFE) method, where feature importance was determined by cumulative weight values. Subsequently, the feature factors selected through machine learning were cross-validated to identify the optimal feature factors for use in further research. A Venn diagram was generated using jvenn (27).

### Model construction and performance evaluation

In this study, we conducted analyses using five classic ML models: the LR classifier, eXtreme Gradient Boosting (XGBoost) classifier, multilayer perceptron (MLP) classifier, SVM classifier, and random forest (RF) classifier. Our methodology divided the dataset into training (80%) and validation (20%) sets. This approach, which is designed to sequentially rotate the test set, enhances the reliability of our results by reducing random variance. For each algorithm, we adopted a rigorous training regimen using fivefold nested cross-validation on the training dataset. The determination of the optimal parameters for each model was facilitated through a comprehensive grid search, the specifics of which are listed in Supplementary Table S2. Building on this foundation, we enhanced our methodology by developing a stacked ensemble model. The model, which integrated three selected basic classifiers, was constructed with LR as the stacking algorithm of the meta-classifier. This integration aimed to enhance the accuracy and generalizability of our results.

The LR classifier is recognized as a classic and commonly utilized model for risk prediction because of its simplicity in model configuration, rapid training speed, and excellent interpretability (28). The XGBoost classifier has emerged as a popular ML algorithm that is renowned for its high performance and flexibility, serving as an effective implementation of the gradient boosting framework (29). The MLP classifier, which is also a classic in the ML domain, employs backpropagation to train the network, calculates the error between the actual and predicted outputs and adjusts the weights by propagating this error back through the system. Thus, it is highly effective for complex problem solving (30). The SVM classifier, which leverages kernel functions, achieves linear separation in high-dimensional space and is recognized for its stability (31). The RF classifier is known for its robustness. It operates by constructing numerous decision trees during training and deriving the class by the mode of the classes of individual trees for classification tasks. This model has demonstrated its effectiveness across a variety of classification problems (32). Stacked ensemble algorithms is a widely applied ensemble learning method that combines basic classifier models to yield predictions with higher accuracy and better generalization capabilities (33).

Model performance was assessed through receiver operating characteristic (ROC) analysis, and the area under the curve (AUC) and 95% confidence interval (CI) were used as the key metrics for evaluating model efficacy. Then, the accuracy, sensitivity, and specificity were calculated. In addition, a calibration curve was used to evaluate the model performance.

All ML processes were carried out using Python 3.7 with several essential libraries: Scikit-learn (1.1.3) for implementing machine learning models, including Logistic Regression with L1 regularization, SVM, and KNN. Pandas (1.2.4) for data manipulation. NumPy (1.20.2) for numerical computations.

## Results

### Characteristics of the cohort

The study cohort included a total of 558 patients, 329 in the EMs group and 229 in the AD group. The age range of the participants in this study ranged from 22 to 67 years. Specifically, the age in the EMs group was 33 (29, 39) years, and in the AD group, it was 34 (30, 39) years. There was no significant difference in age distribution between the two groups (*P* > 0.05), indicating that the hematological indices were comparable. The hematological indices of the participants, which included complete blood cell counts, platelet indices, and markers of coagulation function, are presented in Table 1. Analysis of these indices revealed significant differences in several parameters (*P* < 0.05), including white blood cells (WBCs), mean corpuscular volume (MCV), mean corpuscular hemoglobin concentration (MCHC), red blood cells (RBCs), platelets (PLTs), red cell distribution width (RDW), mean corpuscular hemoglobin (MCH), basophilic granulocytes, plateletcrit (PCT), monocytes, international normalized ratio (INR), activated partial thromboplastin time (APTT), prothrombin time (PT), fibrin degradation product (FDP), and antithrombin III (AT-III), between the EMs and AD groups.

**Table 1:** The baseline characteristics of the participants [M (Q25, Q75)].

Variables	Endometriosis group (*n* = 329)	Adenomyosis group (*n* = 229)	Statistical magnitude	*P*-value
Age, years	33 (29, 39)	34 (30, 39)	−1.227	0.219
White blood cell, *10^9^/L	7.490 (5.480, 10.550)	6.700 (5.150, 9.630)	2.079	0.038
Eosinophilic granulocyte, *10^9^/L	0.060 (0.014, 0.110)	0.060 (0.024, 0.120)	−0.959	0.337
Hematocrit, /	0.347 (0.316, 0.379)	0.349 (0.299, 0.389)	0.155	0.877
Hemoglobin, g/L	114.000 (103.000, 126.000)	114.000 (91.400, 127.000)	1.665	0.096
Mean corpuscular volume, fL	88.600 (84.700, 91.400)	87.000 (80.000, 90.400)	3.489	<0.001
Mean corpuscular hemoglobin concentration, g/L	330.000 (322.000, 337.000)	324.000 (310.000, 332.000)	6.344	<0.001
Mean platelet volume, fL	9.900 (8.800, 10.800)	9.700 (9.000, 10.500)	0.893	0.372
Red blood cell, *10^12^/L	4.010 (3.660, 4.330)	4.130 (3.720, 4.480)	−2.157	0.031
Platelet, *10^9^/L	226.000 (184.000, 273.000)	244.000 (199.000, 290.000)	−2.365	0.018
Red cell distribution width, %	13.100 (12.500, 14.300)	14.600 (13.100, 17.800)	−7.630	<0.001
Neutrophil, *10^9^/L	5.200 (3.300, 8.500)	4.410 (3.200, 7.550)	1.599	0.110
Average hemoglobin volume, pg	29.400 (27.700, 30.400)	28.300 (25.200, 29.900)	5.119	<0.001
Lymphocyte, *10^9^/L	1.410 (1.100, 1.800)	1.380 (1.100, 1.780)	0.563	0.573
Platelet distribution width, fL	16.000 (13.400, 16.500)	15.900 (14.500, 16.500)	0.848	0.396
Basophilic granulocyte, *10^9^/L	0.020 (0.000, 0.030)	0.020 (0.010, 0.030)	−3.086	0.002
Plateletcrit, /	0.220 (0.180, 0.260)	0.240 (0.190, 0.280)	−2.438	0.015
Monocyte, *10^9^/L	0.480 (0.330, 0.630)	0.420 (0.300, 0.580)	2.323	0.020
International Normalized Ratio, /	1.050 (1.000, 1.120)	1.030 (0.970, 1.080)	3.260	0.001
Thrombin time, s	16.200 (15.200, 17.000)	16.300 (15.500, 17.300)	−1.640	0.101
Activated partial thromboplatin time, s	29.600 (26.800, 35.500)	28.000 (25.400, 32.300)	4.422	<0.001
Prothrombin time, s	12.700 (11.900, 13.500)	12.300 (11.600, 13.100)	3.019	0.003
Plasma D-dimer, mg/L	0.700 (0.290, 1.580)	0.549 (0.233, 1.575)	1.052	0.293
Fibrinogen, g/L	2.840 (2.400, 3.540)	2.820 (2.400, 3.460)	0.037	0.971
Fibrin degradation product, ug/ml	3.100 (1.600, 5.830)	2.100 (1.242, 5.600)	2.307	0.021
Antithrombin Ⅲ, %	87.000 (77.773, 95.700)	89.000 (82.500, 96.600)	−2.154	0.031

### Variable filter

**Figure 1:**
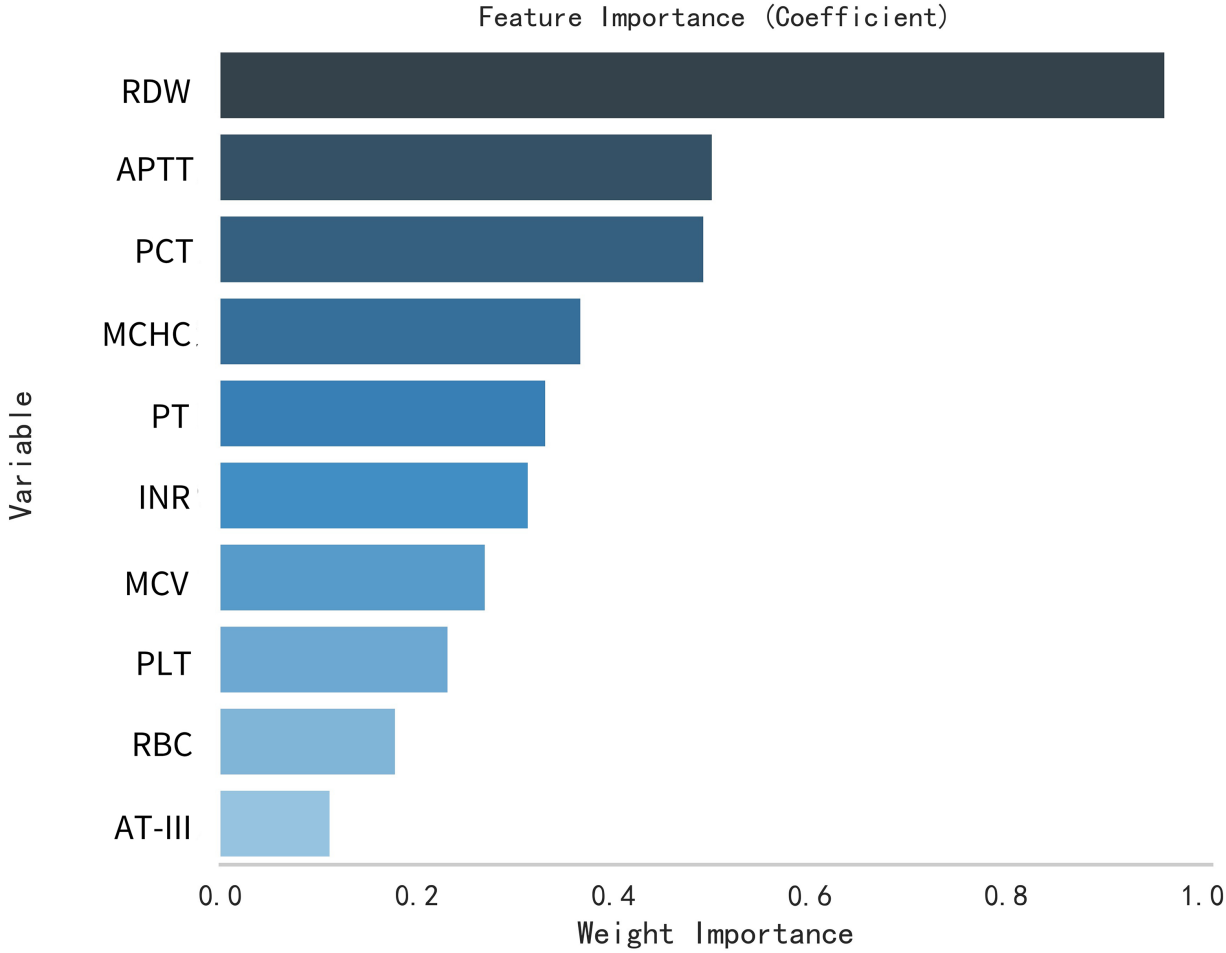
Analysis of feature importance for hematological markers selected using the LR model.

**Figure 2:**
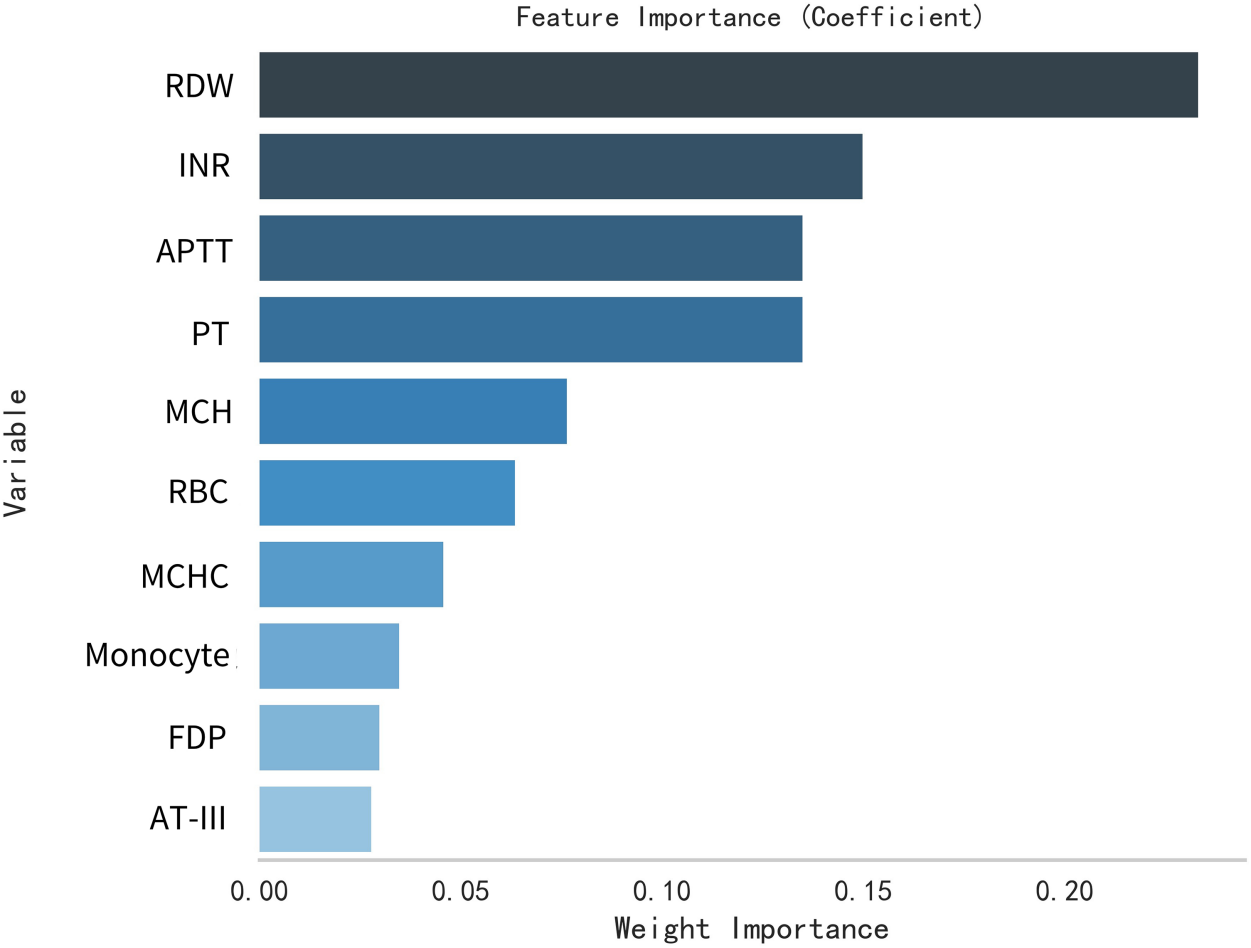
Analysis of feature importance for hematological markers selected using the SVM model.

**Figure 3:**
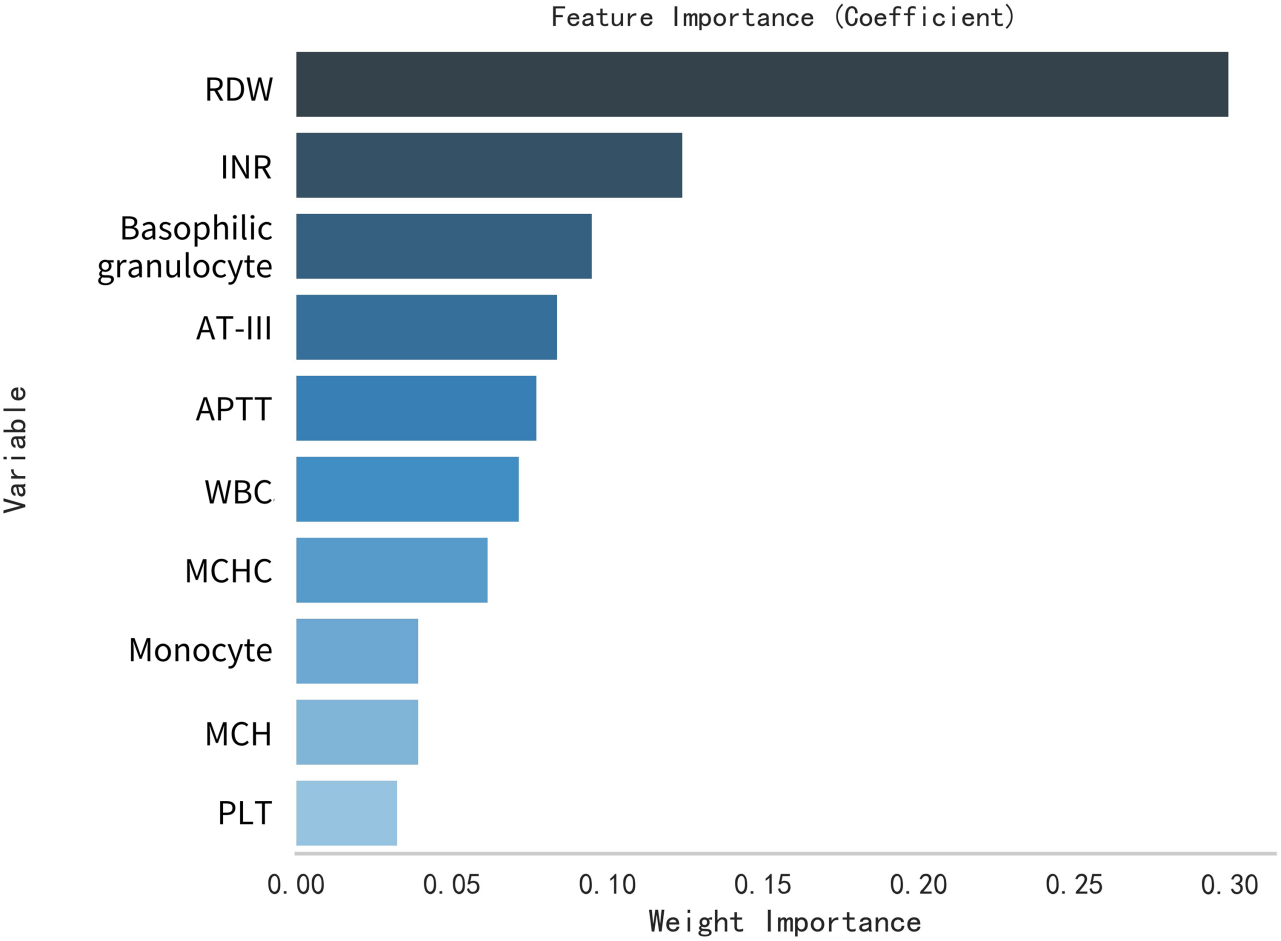
Analysis of feature importance for hematological markers selected using the KNN model.

**Figure 4:**
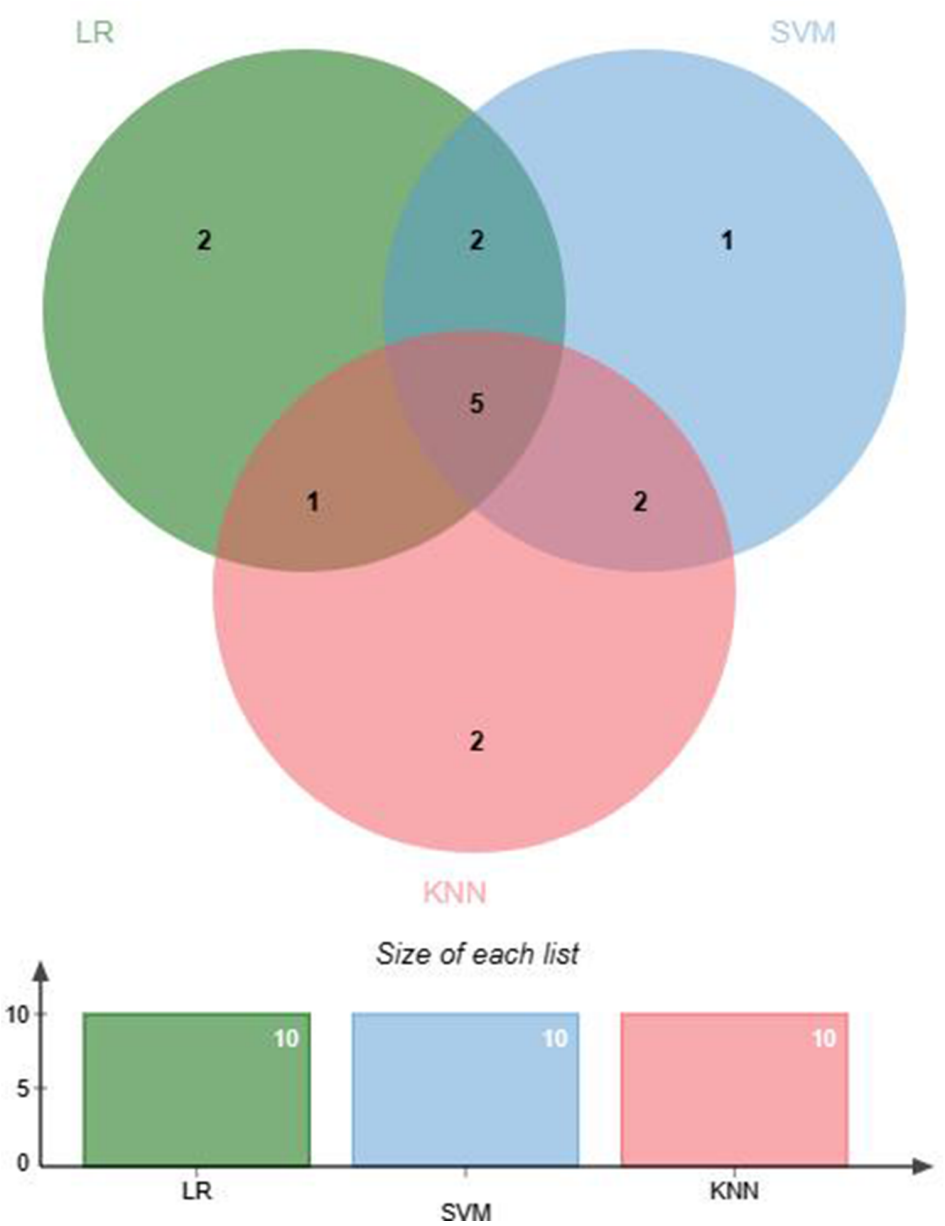
Venn diagram combining feature factor selections from machine learning models.

This study selected differential hematological indices from cohort characteristics for feature factor screening. The LR algorithm of L1 regularization was used to filter important variables. The top ten variables identified were RDW, APTT, PCT, MCHC, PT, INR, MCV, PLTs, RBCs, and AT-III. For further analysis, a SVM classification algorithm was employed, revealing the top ten variables: RDW, INR, APTT, PT, MCH, RBCs, MCHC, monocytes, FDP, and AT-III. Additionally, the KNN classification algorithm was utilized to identify the top ten variables: RDW, INR, basophilic granulocyte, AT-III, APTT, WBCs, MCHC, monocytes, MCH, and PLTs. The importance coefficients of the feature factors identified by these three feature selection methods are illustrated in Figures 1–3. A Venn diagram was constructed to identify the intersection of feature factors derived from the LR, SVM, and KNN algorithms. The intersection targets for the three datasets were RDW, APTT, MCHC, INR, and AT-III (Figure 4).

### ML model evaluation

**Figure 5:**
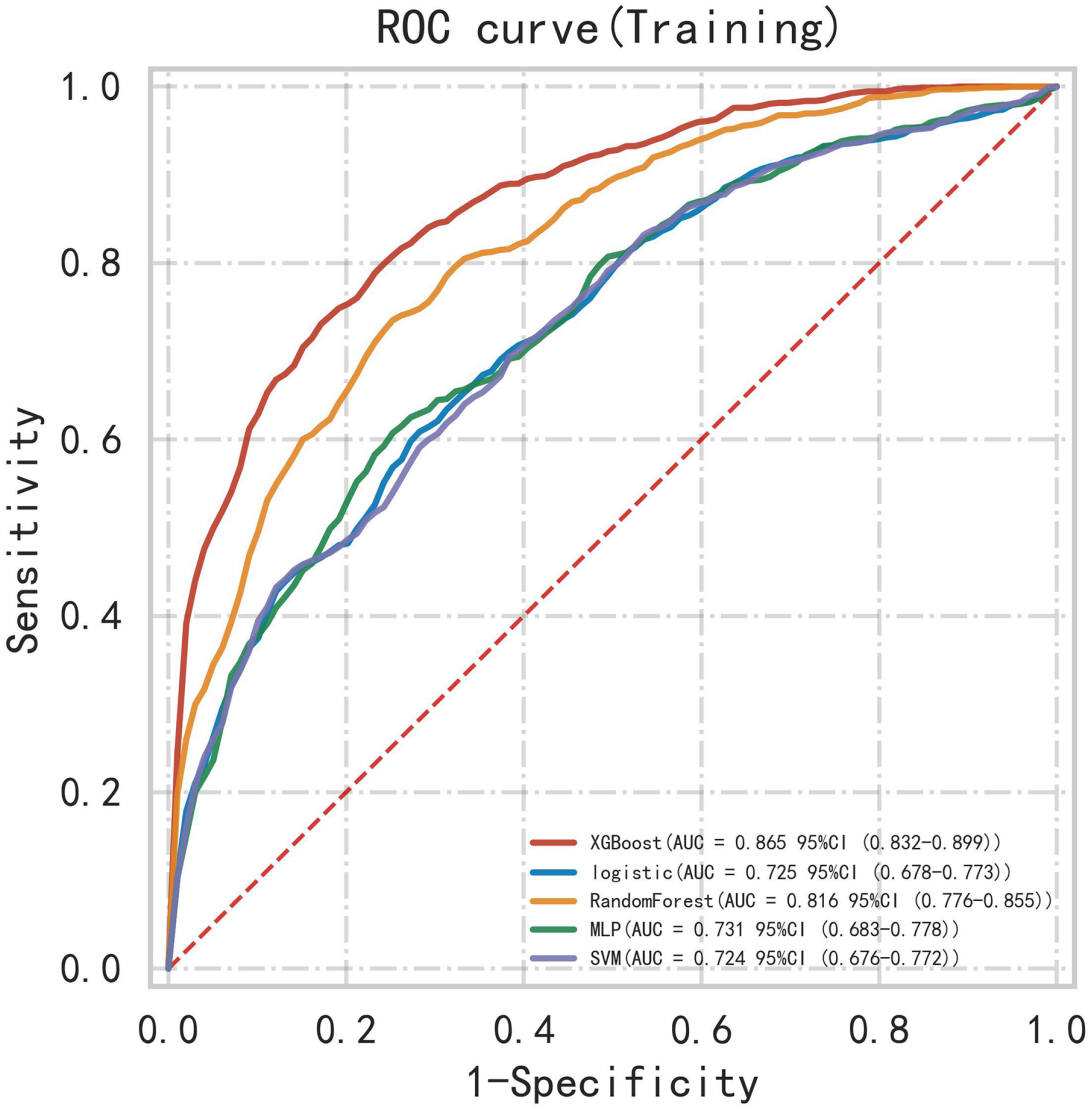
ROC curve for multiple classic model classifications of the training set.

**Figure 6:**
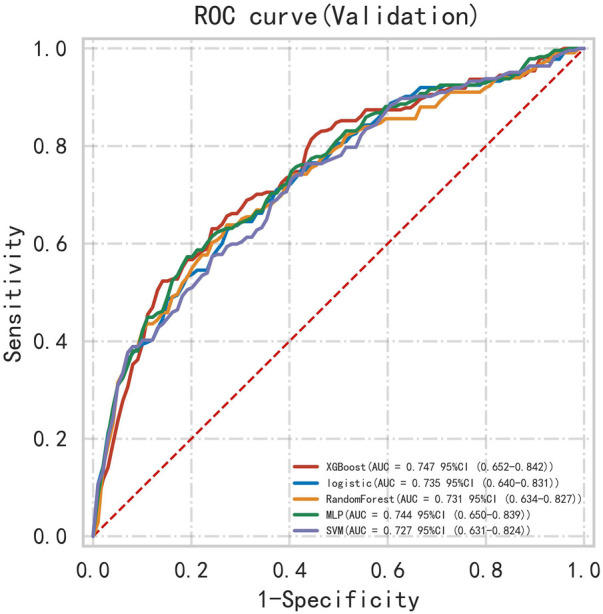
ROC curve for multiple classic model classifications of the validation set.

**Figure 7:**
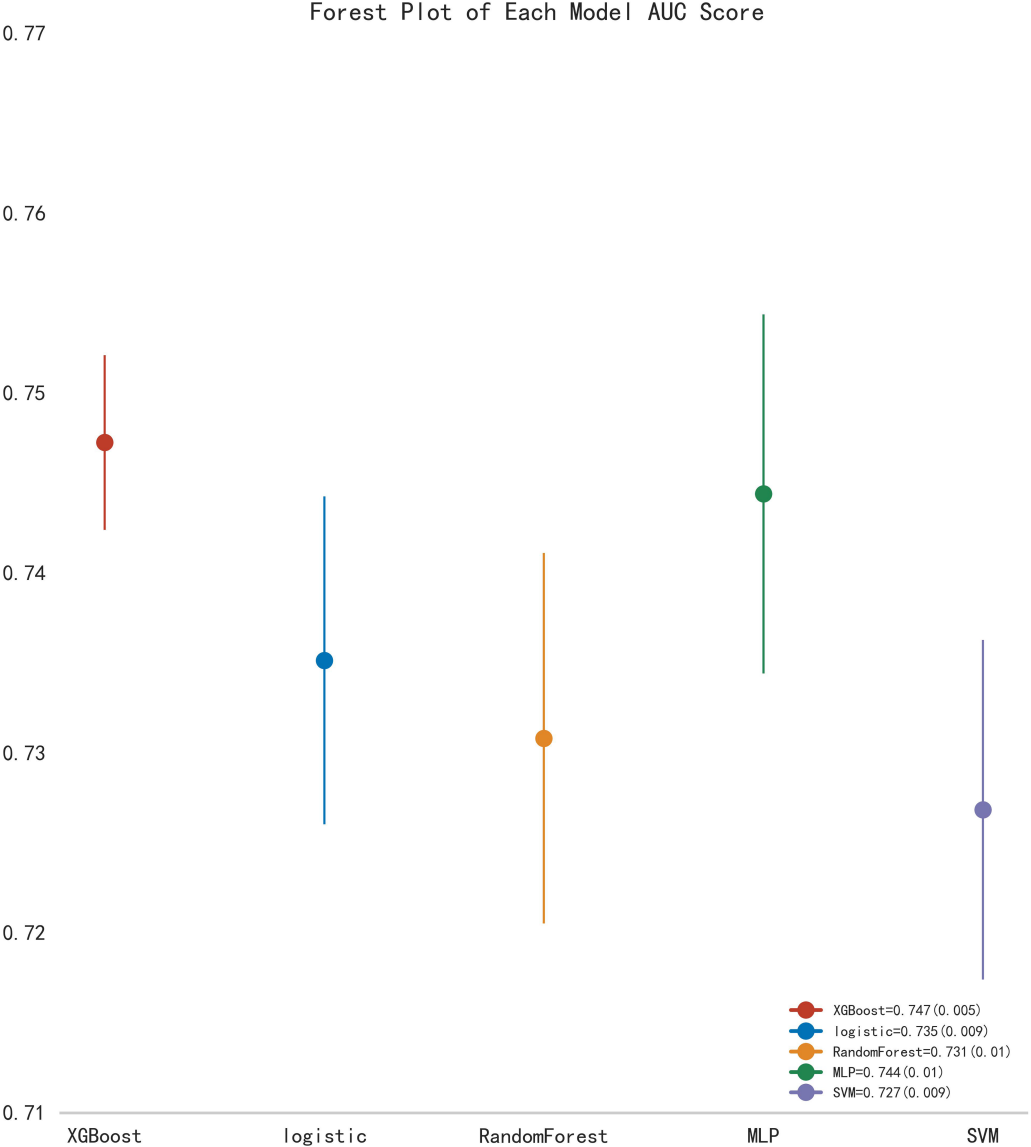
Forest plot of AUC scores for multiple classic model classifications.

First, five classic ML models were developed and validated, and the model performances are listed in Tables 2, 3 and Figures 5–7. Among these, the model with the best performance on the training set was the XGBoost classification model (AUC) = 0.865, 95% CI: 0.832–0.899), followed by the RF classification model (AUC = 0.816, 95% CI: 0.776–0.855), the MLP classification model (AUC = 0.731, 95% CI: 0.683–0.778), the LR classification model (AUC = 0.725, 95% CI: 0.678–0.773), and the SVM classification model (AUC = 0.724, 95% CI: 0.676–0.772). The XGBoost classification model also showed the best performance in the validation set (AUC = 0.747, 95% CI: 0.652–0.842), followed by the MLP classification model (AUC = 0.744, 95% CI: 0.650–0.839), the LR classification model (AUC = 0.735, 95% CI: 0.640–0.831), the RF classification model (AUC = 0.731, 95% CI: 0.634–0.827), and the SVM classification model (AUC = 0.727, 95% CI: 0.631–0.824).

**Table 2:** Performance metrics of machine learning models on the training cohort.

Model	AUC (95% CI)	Accuracy	Sensitivity	Specificity
LR classifier	0.725 (0.678–0.773)	0.664	0.695	0.645
XGBoost classifier	0.865 (0.832–0.899)	0.788	0.767	0.806
MLP classifier	0.731 (0.683–0.778)	0.694	0.623	0.747
SVM classifier	0.724 (0.676–0.772)	0.666	0.669	0.666
RF classifier	0.816 (0.776–0.855)	0.746	0.753	0.745

**Table 3:** Performance metrics of machine learning models on the validation cohort.

Model	AUC (95% CI)	Accuracy	Sensitivity	Specificity
LR classifier	0.735 (0.640–0.831)	0.643	0.684	0.710
XGBoost classifier	0.747 (0.652–0.842)	0.693	0.803	0.652
MLP classifier	0.744 (0.650–0.839)	0.702	0.658	0.770
SVM classifier	0.727 (0.631–0.824)	0.627	0.699	0.688
RF classifier	0.731 (0.634–0.827)	0.684	0.665	0.741

**Figure 8:**
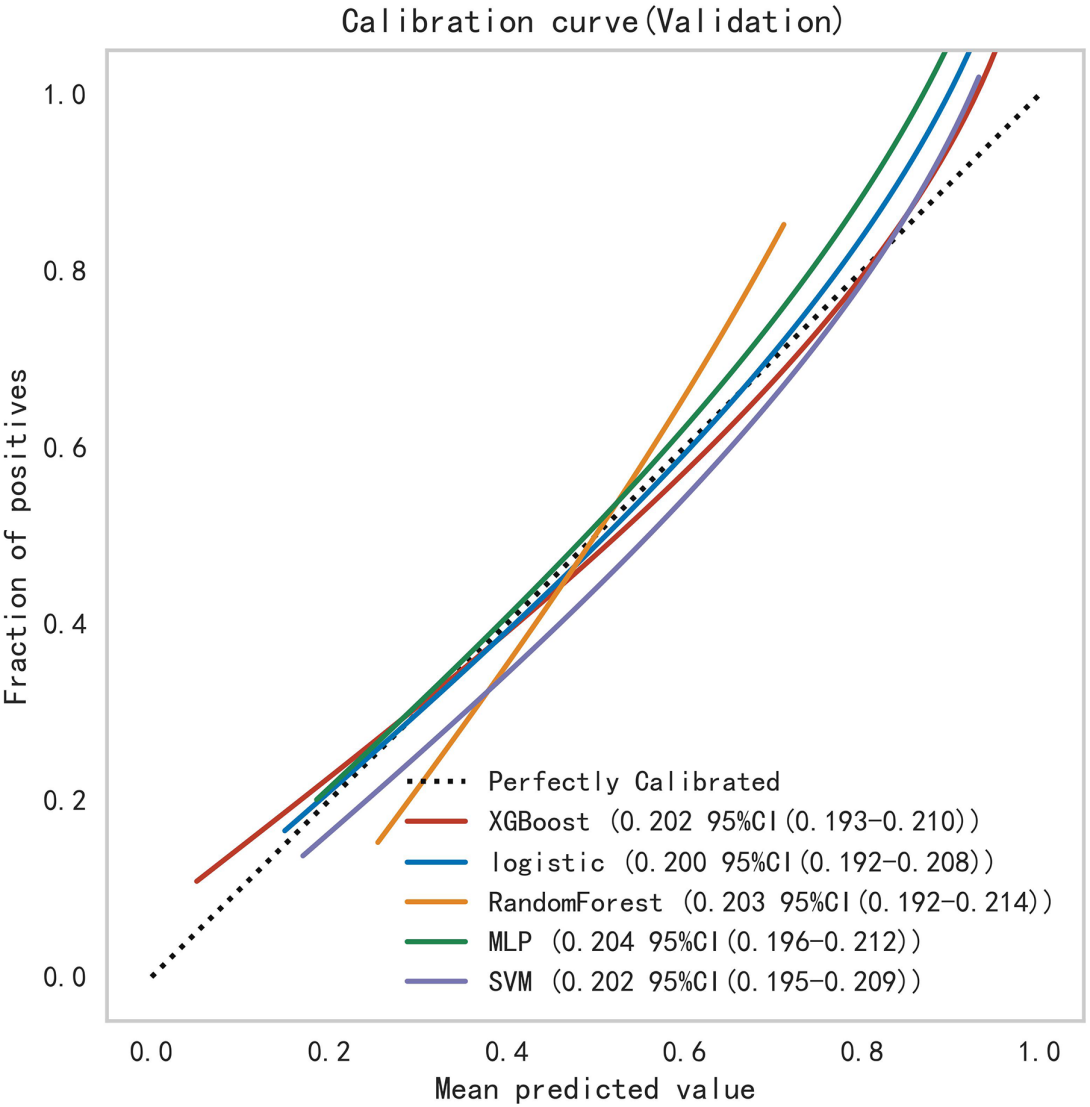
Calibration curves for the validation sets of multiple models.

Subsequently, the ROC curves of the five ML methods were tested using the DeLong test. The results indicated that there was no statistically significant difference in the ROC curves of the aforementioned machine learning models (*P* > 0.05), as shown in Table 4. Calibration curves of the validation set for multiple models (Figure 8) demonstrated that the predicted probabilities of the five machine classification models were close to the actual probabilities.

**Table 4:** Delong detection results for multiple model classification.

Machine learning model		LR classifier	RF classifier	SVM classifier	MLP classifier
XGBoost classifier	*Z*-value	0.319	0.809	0.506	0.524
	*P*-value	0.769	0.504	0.626	0.634
MLP classifier	*Z*-value	0.966	0.949	1.159	
	*P*-value	0.430	0.487	0.382	
SVM classifier	*Z*-value	0.912	0.857		
	*P*-value	0.472	0.452		
RF classifier	*Z*-value	0.770			
	*P*-value	0.520			

**Figure 9:**
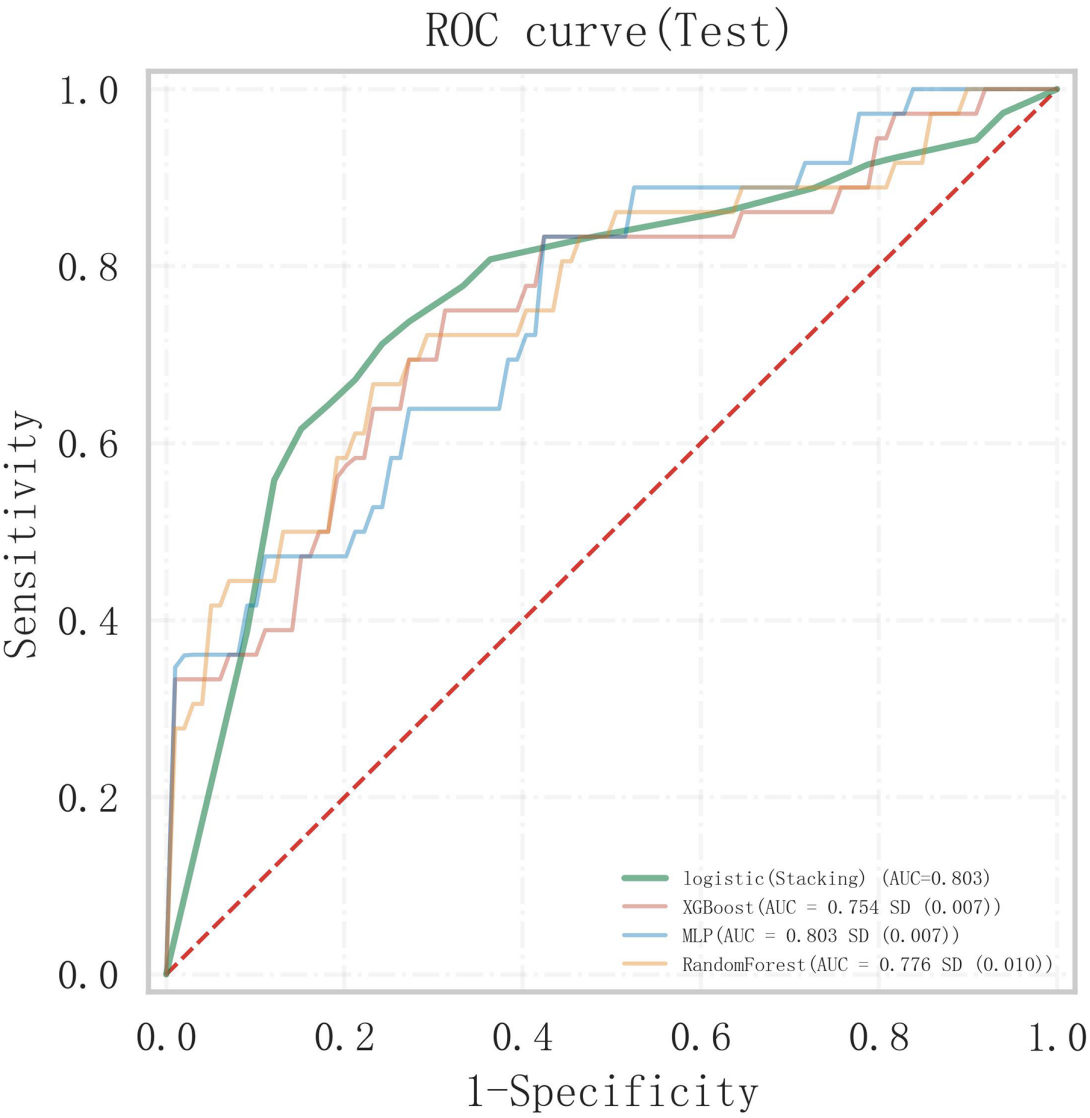
Comparison of ROC curves between the stacked ensemble machine learning model and basic machine learning models.

A stacked ensemble model was constructed by selecting the three best-performing base learners (XGBoost, RF, and MLP). The stacked ensemble model was built on the second-layer LR meta-model based on the first layer of base learners, with the following model parameters: regularization factor: 1, number of iterations: 100, type of regularization: l2, convergence metric: 0.0001. Compared to XGBoost (AUC = 0.754, 95% CI: 0.647–0.860; Specificity = 0.669), RF (AUC = 0.778, 95% CI: 0.673–0.883; Specificity = 0.846), and MLP (AUC = 0.802, 95% CI: 0.698–0.906; Specificity = 0.863), the stacked ensemble model achieved an AUC = 0.803 (95% CI: 0.701–0.904; Specificity = 0.875), as detailed in Table 5 and Figure 9. These results showed that the ensemble model outperformed the individual models in terms of classification accuracy for EMs and AD, with improved performance and stronger generalizability.

**Table 5:** The performance metrics of the comparison between the stacked ensemble machine learning model and classical machine learning models.

Model	AUC (95% CI)	Accuracy	Sensitivity	Specificity
Stacked ensemble model	0.803 (0.701–0.904)	0.774	0.667	0.875
XGBoost classifier	0.754 (0.647–0.860)	0.693	0.711	0.669
MLP classifier	0.802 (0.698–0.906)	0.774	0.706	0.863
RF classifier	0.778 (0.673–0.883)	0.721	0.656	0.846

## Discussion

### Contextualizing with previous research

EMs and AD are distinct but closely related conditions involving the presence of endometrial tissue outside the uterine lining. EMs is a chronic inflammatory disease that significantly impairs quality of life, often causing cyclic pain and infertility. AD is characterized by the invasion of endometrial tissue into the myometrium, leading to myometrial hypertrophy (34, 35). Currently, the definitive diagnosis of both conditions relies on invasive surgical or pathological examination, which is not always feasible. This underscores the need for non-invasive diagnostic methods to improve patient management (36). Recent studies have explored the use of ML for non-invasive diagnosis of EMs and AD. Guerriero et al. (37) used LR to differentiate EMs from AD based on ultrasound imaging. Balica et al. (38) employed five ML models (Xception, Inception-V4, ResNet50, DenseNet, and EfficientNetB2) to assist in ultrasound diagnosis, achieving an AUC of 90% and an accuracy of 80%. Raimondo et al. (39) developed a deep learning model for ultrasound-based diagnosis of AD, noting its potential to reduce overdiagnosis. However, ultrasound diagnosis is heavily dependent on the examiner's expertise, which can result in missed or incorrect diagnoses, particularly in early or deep pelvic lesions. Our study offered a novel approach by integrating readily accessible peripheral blood and coagulation markers into a stacked ensemble ML model. This approach enhanced the accuracy and reliability of differentiating between EMs and AD. It addressed a critical gap in the non-invasive diagnosis of these conditions and contributes to early diagnosis and personalized treatment strategies.

### Identification of key hematological and coagulation markers

In this study, we demonstrated the feasibility and sensitivity of applying ML methods to screen for characteristic factors, including complete blood counts, platelet indices, and coagulation markers, that could differentiate between EMs and AD. Five features were selected as hematological indicators for assessing EMs and AD and were identified as key potential factors for discriminating between the two diseases. Furthermore, based on five classic ML models, we identified the three with the best performance for the construction of a stacked ensemble model. The stacked model emerged as the optimal model for distinguishing between EMs and AD (AUC = 0.803, 95% CI: 0.701–0.904).

Considering the computational resources wasted on redundant and irrelevant features within the original feature set during model training and prediction (40), we employed three ML methods (LR, SVM, and KNN) for feature selection and performed cross-validation to retain useful feature factors. L1-regularized LR improves model efficiency and generalization by reducing the number of features, as it retains only those features that significantly contribute to predictions while eliminating noise (41). In contrast, SVM and KNN excel at handling non-linear relationships. When using RFE, they can recursively eliminate the least impactful features, allowing the models to focus on the most relevant aspects of the data, thereby enabling faster and more accurate predictions (42). The combined use of the three methods effectively leverages the strengths of different models. Precise feature factor selection not only enhances model performance but also increases model transparency and interpretability (43). More intriguingly, this study identified key features, particularly RDW, MCHC, APTT, INR, and AT-III, through machine learning techniques, highlighting the hematological differences between patients with EMs and patients with AD. These findings underscore the potential differences in bleeding and coagulation between the two conditions.

### Clinical relevance of hematological findings

Our study revealed that, as significant characteristic factors in the peripheral blood for these two diseases, the RDW in patients with AD was greater than that in patients with EMs, and the MCHC in patients with AD was lower than that in patients with EMs. RDW represents the standard deviation or coefficient of variation percentage of RBC volume, indicating significant size disparities in certain anemias. An increase in RDW reflects a severe disruption in erythrocyte homeostasis, including impaired erythrocyte production and abnormal erythrocyte survival (44). The MCHC is a critical indicator among the erythrocyte parameters that often suggests anemia when decreased (45). Dugdale et al. (46) reported a negative correlation between RDW and hemoglobin concentration over several months. An increase in RDW precedes a clinically significant decrease in hemoglobin levels by weeks; therefore, RDW is recommended as a valuable routine marker for the early detection of iron deficiency anemia. AD patients often exhibit clinical manifestations of increased menstrual flow (7), but EMs patients do not. This may explain the differences in the RDW and MCHC, suggesting a greater likelihood of bleeding and a predisposition to anemia in AD patients.

In our research, we identified three specific coagulation factors related to clotting: APTT, INR, and AT-III. The APTT and INR in the AD cohort were lower than those in the EMs cohort, and the AT-III levels were greater in the AD cohort. The APTT is a critical parameter for coagulation that is commonly used to predict bleeding tendencies and hypercoagulable states. A decrease in APTT is associated with hypercoagulability, indicated by an increase in thrombin generation and a greater risk of thrombosis (47). INR is a standardized PT that adjusts for variations in coagulation activator reagents, allowing the PT values measured by different laboratories and reagents to be comparable. A lower INR suggests an increased risk of thrombosis (48). This study demonstrated that patients with AD have a greater hypercoagulable state and greater thrombotic risk than patients with EMs. Lin et al. (49) reported that APTT is decreased in AD patients, indicating a hypercoagulable state. Yang et al. (24) reported a significant decrease in APTT among AD patients with thrombosis. A study indicated that shorter APTT in EMs patients might be related to a potential hypercoagulable state associated with the disease, and the role of the local coagulation system in the disease pathogenesis cannot be excluded (50). Lin et al. (49) also showed a negative correlation between coagulation markers and hemoglobin in AD patients with anemia. Anemia can affect coagulation parameters and increase the risk of thrombus formation (51). Yamanaka et al. (52) suggested that the coagulation dysfunction caused by AD could be a possible reason for thrombosis formation and menorrhagia. Menorrhagia can lead to anemia, further promoting a hypercoagulable state and possibly leading to thrombosis in a vicious cycle (49). Based on these studies, we hypothesize that menorrhagia-induced anemia and subsequent hypercoagulable changes are the reasons for the specific differences in coagulation markers between patients with AD and patients with EMs, but the related underlying physiological mechanisms require further investigation. Interestingly, AT-III levels were greater in the AD cohort than in the EMs cohort, which seems inconsistent with the trends in APTT and INR. EMs is an inflammatory disease characterized by increased expression of inflammatory and angiogenic factors (53). The peritoneal fluid of patients with EMs contains high levels of macrophages and immune cells, which secrete cytokines, angiogenic factors, and growth factors (54–56). AT-III is a nonvitamin K-dependent protease that regulates coagulation and inhibits inflammation within the endothelium (57, 58). Therefore, we speculate that AT-III is activated in the inflammatory response caused by the abnormal growth of endometrial tissue in EMs patients and plays an anti-inflammatory role by increasing its consumption within the vasculature.

### Evaluation of the stacked ensemble model

Compared with traditional clinical diagnostic prediction models, the stacked ensemble model based on multiple machine learning algorithms successfully developed by us exemplifies a powerful and flexible strategy in machine learning. It integrates numerous learners and strategically adjusts using a meta-learner. Thus, this model achieves exceptional classification performance and significantly enhances the ability to generalize to new data (59). While stacked ensemble models are widely used in other areas, their application to the noninvasive diagnosis of EMs and AD has not been extensively explored. This study applies a stacked ensemble model in this specific clinical context, contributing to the understanding of its potential in this area. Our research demonstrates that the stacked ensemble model outperforms traditional models by achieving a higher AUC (0.803) and excelling in specificity (0.875). This consistent advantage underscores its potential for more accurate and reliable differentiation between EMs and AD. The model's balanced performance in both accuracy and specificity highlights its robustness and suitability for clinical applications where precision and reliability are crucial.

### Limitations and future directions

Furthermore, peripheral blood and coagulation markers are cost-effective and readily available indicators. Thus, our study was able to screen for early classification predictions of EMs and AD without the need for invasive diagnostic methods. This advancement aids clinicians in the early identification of high-risk patients and in taking timely measures to address relevant risk factors, thereby devising more rational diagnostic and therapeutic plans. It is important to note the limitations of our study. Our research was conducted as a single-center study, and further research will need to collect data from patients across different countries or regions to enhance the generalizability of the research findings. While it is effective in differentiating between EMs and AD, further development and validation would be required to extend its application to a broader diagnostic context. Expanding the model's capability to identify these conditions among a wider range of possible diagnoses could significantly advance computer-assisted diagnostic tools.

## Conclusion

In this study, we developed a model based on stacked ensemble machine learning algorithms for the classification prediction of patients with EMs and AD. The results indicate that RDW, MCHC, PTT, INR, and AT-III are significant characteristic factors for distinguishing between EMs and AD. The model demonstrates excellent classification prediction accuracy and clinical utility, enabling the early, convenient, and noninvasive identification of patients with EMs and AD. The model also assists in clinical decision-making, supporting physicians in implementing personalized treatment plans.

## Data Availability

The original contributions presented in the study are included in the article/Supplementary Material, further inquiries can be directed to the corresponding authors.
