# Supplementary material for: A stacked machine learning-based classification model for endometriosis and adenomyosis: a retrospective cohort study utilizing peripheral blood and coagulation markers
Source: Front Digit Health. 2024 Sep 10;6:1463419. doi: 10.3389/fdgth.2024.1463419 (PMC11428011; doi:10.3389/fdgth.2024.1463419)
Supplement: Supplementary file 1 [file Table1.docx]

**Supplementary table 1. Parameters of machine learning models for features selection.**

| Method | Parameters |
| --- | --- |
| LR classification | regularization type: 'L1', number of iterations: 100, convergence metric: 0.0001, regularization factor: 1.3987131 |
| SVM classification | regularization factor: 1.0, kernel type: 'rbf', convergence metric: 0.001 |
| KNN classification | number of neighbors: 6, weight type: 'uniform' |

**Supplementary table 2. The parameters of the five candidate models.**

| Method | Parameters |
| --- | --- |
| LR classification | C: 10, maximum iterations: 100, solver: 'liblinear', tolerance: 1e-09 |
| XGBoost classifier | learning rate: 0.5, maximum depth: 1, minimum child weight: 1, regularization lambda: 0.1 |
| MLP classifie | activation: 'logistic', hidden layer sizes: (200, 200), maximum iterations: 150 |
| SVM classifier | C: 10, kernel: 'linear', tolerance: 1e-06 |
| RF classifier | criterion: 'gini', maximum depth: 10, minimum impurity decrease: 0.01, number of estimators: 200 |
